# External validity of a prediction rule for residual mass histology in testicular cancer: an evaluation for good prognosis patients

**DOI:** 10.1038/sj.bjc.6600759

**Published:** 2003-03-18

**Authors:** Y Vergouwe, E W Steyerberg, R de Wit, J T Roberts, H J Keizer, L Collette, S P Stenning, J D F Habbema

**Affiliations:** 1 Center for Clinical Decision Sciences, Department of Public Health, Ee20-42, Erasmus MC, PO Box 1738, 3000 DR Rotterdam, The Netherlands; 2 Department of Internal Oncology, Erasmus MC - Daniel, PO Box 5201, 3008 AE, Rotterdam, The Netherlands; 3 Northern Centre for Cancer Treatment, Westgate Road, Newcastle upon Tyne NE4 6BE, UK; 4 Department of Clinical Oncology, Leiden University Medical Center, Leiden, The Netherlands; 5 European Organisation for Research and Treatment of Cancer, Data Center, Avenue E Mounier 83, 1200 Brussels, Belgium; 6 Medical Research Council, Clinical Trials Unit, 222 Euston Road, London NW1 2DA, UK

**Keywords:** testis, residual neoplasms, histology, statistical models, validity

## Abstract

We assessed the external validity of a prediction rule for nonseminomatous testicular cancer patients. The rule was developed to predict the probability of retroperitoneal metastases being benign (only necrosis/fibrosis) after chemotherapy treatment. Patients with a high probability of benign residual masses might be offered surveillance as opposed to patients with a low probability, who should undergo retroperitoneal lymph node dissection (RPLND). We compared the observed histology with the predicted probability in 105 patients with good prognosis germ cell cancer who underwent RPLND between 1995 and 1998. We found that predicted probabilities higher than 5% were in good agreement with the observed frequencies of benign masses. The area under the receiver operating characteristic curve was 0.76, suggesting that the rule could reasonably discriminate between benign masses and tumour. However, nearly all predicted probabilities (*n*=101) were lower than 70%, which might be considered as the lowest value at which surveillance offers a reasonable alternative to RPLND. Further, 35% of patients currently under surveillance (84 out of 241) had predicted probabilities lower than 70%. In conclusion, the clinical relevance of the prediction rule was limited for the patients who underwent RPLND; use of the rule would change the policy from RPLND to surveillance in only a few. On the other hand, the rule might support selection of patients for RPLND, who currently are under surveillance.

Computer tomography (CT) often shows small remnants of retroperitoneal masses after chemotherapy for metastatic nonseminomatous testicular cancer (Peckham, 1988). The histology of the residual masses may be benign (entirely necrotic/fibrotic), or may contain tumour elements (mature teratoma or viable cancer cells). Resection of a totally benign mass has no therapeutic value and should preferably not be performed. Most resection policies consider only one prognostic factor to predict the histology of residual masses, that is, mass size after chemotherapy (Jansen *et al*, 1991; Mead *et al*, 1992). Masses smaller than or equal to 10 mm are generally not resected, although more aggressive approaches have been proposed (Gelderman *et al*, 1986; Fosså *et al*, 1992).

Mass size as a single prognostic factor has limited predictive power to discriminate benign histology from tumour. Some small masses containing tumour are left unresected and larger benign masses are unnecessarily removed. A distinction based on several prognostic factors has the potential to classify masses more accurately as benign or tumour (Donohue *et al*, 1987; Fosså *et al*, 1992). Therefore, a clinical prediction rule has been developed that incorporates six well-known prognostic factors, that is, the *Re*sidual *Hi*stology in *T*esticular Cancer (ReHiT) prediction rule (Steyerberg *et al*, 1995). It estimates the probability that a residual mass is completely benign. The predicted probability may support the treating physician in deciding whether a residual mass should be resected or not.

Before any wide use of a prediction rule can be encouraged, its ability to produce accurate predictions for patients from different but plausibly related populations (‘transportability’) needs to be assessed (Justice *et al*, 1999). The ReHiT prediction rule was developed for good, intermediate, and poor prognosis patients according to the International Germ Cell Consensus Classification (IGCCG, 1997) on the basis of data from patients from six European and US study groups (development population), who were predominantly treated in the 1980s with cisplatin-based chemotherapy. Patients with a good prognosis (56% of all nonseminomas) have an expected 5-year progression-free survival probability of 89% (IGCCG, 1997). In this group, particularly, it is important to minimise the therapeutic burden; any unnecessary treatment such as resection should be avoided. We therefore studied the transportability of the prediction rule to good prognosis patients treated in the 1990s. We were particularly interested in the clinical relevance of the prediction rule, that is, its ability to support decision-making for patients after chemotherapy.

## PATIENTS AND METHODS

**Figure 1:**
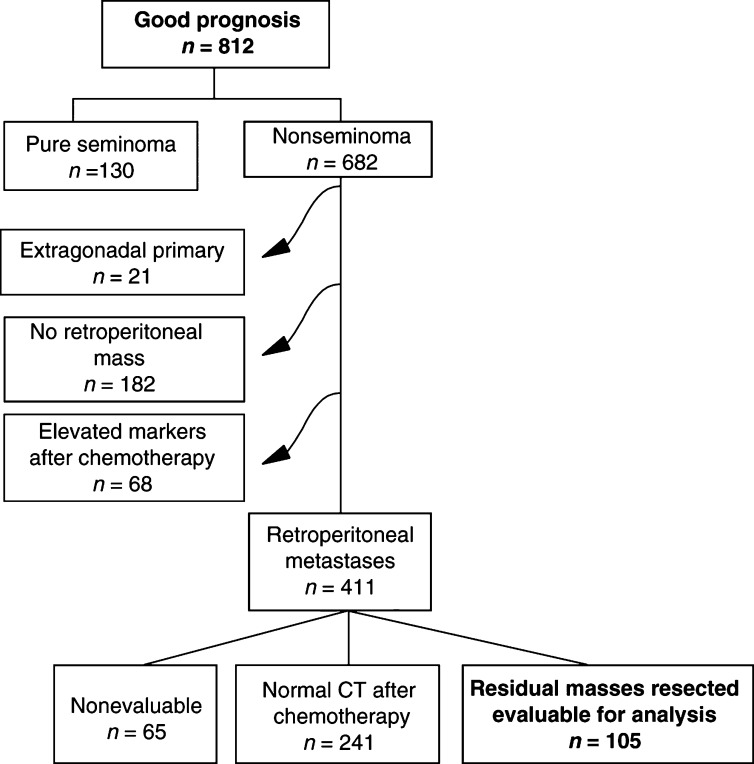
Selection of 105 patients from the EORTC-30941/MRC-TE20 study for whom the prediction rule could be validated.

Patients participated in an EORTC/MRC trial of the genitourinary group (EORTC-30941/MRC-TE20), which compared three cycles of bleomycin, etoposide, cisplatin (3BEP) with four cycles (3BEP–1EP) and the administration of BEP over 5 days with 3 days (de Wit *et al*, 2001). A total of 812 good prognosis patients were enrolled between March 1995 and April 1998 (Figure 1

). The present analysis included only nonseminomas (*n*=682), which are defined as good prognosis disease when the site of the primary tumour is not mediastinal, no nonpulmonary visceral metastases are present, and the marker levels are good, that is, α-fetoprotein (AFP) and human chorionic gonadotropin (HCG) below 1000 ng ml^−1^ and lactate dehydrogenase (LDH) below 1.5 × upper limit of the normal value (IGCCG, 1997). Patients with an extragonadal primary site (*n*=21), patients having no retroperitoneal metastasis (*n*=182), and patients with elevated markers after chemotherapy (*n*=68) were excluded from the analysis. Out of the remaining 411 patients, 306 patients with a prechemotherapy retroperitoneal metastasis did not undergo retroperitoneal lymph node dissection (RPLND), either because the CT was considered to be normal following chemotherapy (*n*=241) or for other reasons (*n*=65, e.g. uncompleted chemotherapy). This meant that 105 patients were analysed for the relation between the predicted probabilities and the observed histologies (validation population); 241 patients were analysed for the predicted probabilities only. Histological findings at RPLND were classified as benign or tumour. Lesions classified as benign contained only necrotic or fibrotic elements, while tumour contained mature teratoma or viable cancer cells.

The prediction rule was developed in 544 patients and was described in detail before (Steyerberg *et al*, 1995). The following patient characteristics are needed to calculate the probability of benign histology: the absence/presence of teratoma elements in the primary tumour, determined as teratoma differentiated (TD) or malignant teratoma intermediate (MTI); prechemotherapy levels of the serum markers AFP, HCG and LDH; maximal transversal mass size measured on CT before and after chemotherapy.

The exact formula is:


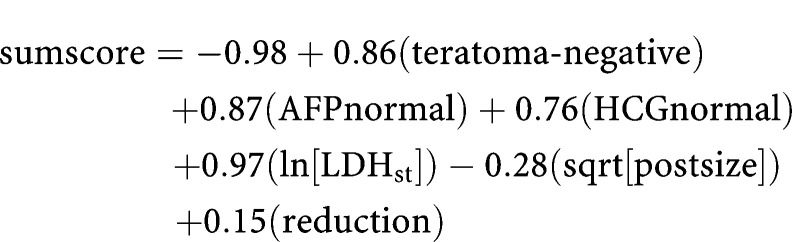


The variables teratoma-negative, AFPnormal, and HCGnormal are 1 if true and 0 if false. Ln[LDH_st_] is the natural logarithm of LDH/upper limit of the normal value range, sqrt[postsize] is the square root of postchemotherapy transverse diameter expressed in millimetres, and reduction is the reduction (per 10%) in mass size during chemotherapy: ((presize−postsize)/presize)*10. The probability of benign histology is calculated with the formula: probability=1/(1+e^−sumscore^).

This complex formula has been transformed into a score chart (Steyerberg *et al*, 1995) for easy estimation of the predicted probability. The value of each variable corresponds to a number of points and the total number of points corresponds directly to the predicted probability. The formula is also implemented in a spreadsheet, which is available in the public domain (ReHiT spreadsheet, http://www.eur.nl/fgg/mgz/software.html).

Missing predictor values (2% of all required values) were imputed based on the correlation with the other predictor variables (He and Shen, 1997). The statistical performance of the prediction rule was studied with respect to calibration and discrimination. Calibration refers to the agreement between the predicted probabilities and the observed frequencies. Calibration was studied graphically (Harrell, 1997) and tested with the Hosmer–Lemeshow test for external validation (Hosmer and Lemeshow, 1989). Discriminative ability, that is, whether the relative ranking of individual predictions is in the correct order, was determined with the area under the receiver operating characteristic (ROC) curve (ROC area) (Harrell *et al*, 1982). The ROC area represents the likelihood that a patient with a benign mass has a higher predicted probability of benign histology than a patient with tumour for a random pair of patients with different histological masses.

To classify masses as benign or tumour using the prediction rule, we applied a threshold value of 70% (Steyerberg *et al*, 1999a). Masses with predicted probabilities higher than 70% were considered benign; masses with probabilities lower than 70% were considered to contain tumour. Using the threshold value, we could study the clinical relevance of the prediction rule for the current population. Clinical relevance was expressed as the proportion of patients, who would receive an alternative treatment, if the prediction rule was applied (i.e. surveillance instead of RPLND).

Calculations were performed with SAS version 6.12 and S-plus version 4.5 software, using the *Hmisc* and *Design* library (Harrell, 1997).

## RESULTS

Table 1

**Table 1:** Distribution of the characteristics of nonseminomatous testicular cancer patients undergoing resection; n (%)

**Patient characteristics**	**Development population *n*=544**	**Validation population *n*=105**
**Predictors**		
*Primary tumour histology*		
Mature teratoma negative	252 (46)	56 (53)
*Prechemotherapy AFP level*		
Normal	186 (34)	33 (31)
*Prechemotherapy HCG level*		
Normal	205 (38)	50 (48)
*Prechemotherapy LDH level*		
Normal	151 (28)	76 (72)
*Postchemotherapy mass size (mm)*		
0–10	165 (30)	8 (8)
11–20	124 (23)	40 (38)
21–50	139 (26)	40 (38)
>50	116 (21)	17 (16)
*Change in mass size during chemotherapy*		
⩾70% reduction	161 (30)	6 (6)
0–69% reduction	341 (63)	67 (64)
1–24% progression	10 (2)	13 (12)
⩾25% progression	32 (6)	19 (18)
		
**Outcome**		
*Worst residual histology*		
Benign	245 (45)	27 (26)
Tumour	299 (56)	78 (75)

shows the distributions of patient characteristics in the development population and in the validation population. Merely 26% (27 out of 105) of the patients in the validation population, which only contained patients with good prognosis disease, had totally benign residual masses. The distributions of the prechemotherapy levels of AFP and HCG and of the histology of the primary tumour were similar across the populations. The validation population contained a far greater number of patients in whom LDH level was normal (72% *vs* 28%), which follows from the definition of good prognosis; LDH level should be less than 1.5 times the upper normal value. The postchemotherapy mass size was larger than 10 mm in 92% of all patients. A very large reduction in mass size during chemotherapy (⩾70%) was seen in only 6% of the validation population.

Figure 2

**Figure 2:**
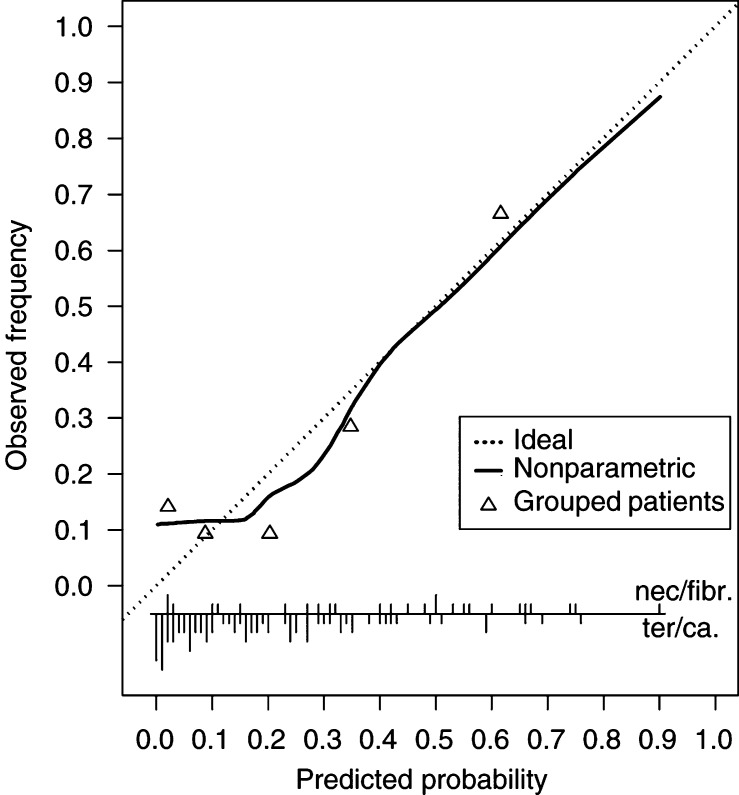
Calibration curve of the prediction rule in patients of the EORTC-30941/MRC-TE20 study. Vertical lines at the bottom indicate the distribution of the predicted probabilities; lines upwards represent patients with a benign mass, lines downwards patients with tumour. Triangles indicate the frequency of benign masses grouped per quintile of predicted probabilities. The solid line shows the relation between predicted probabilities and observed frequencies. Ideally, this line equals the dotted line.

shows the calibration of the prediction rule. The ideal curve represents equality of predicted probabilities and observed frequencies. More than 80% of all patients had predicted probabilities for benign histology smaller than 50%, which is in agreement with the low proportion of patients who actually had benign masses (26%). The Hosmer–Lemeshow test for external validation indicated a poor fit (*P*=0.001). This was caused by three out of 22 patients with a benign histology, while predicted probabilities for benign histology were below 5%. The fit was satisfactory, when these three patients were excluded. The ROC area was 0.76 (95% confidence interval: 0.65–0.88), which indicates reasonable discrimination.

At the threshold value of 70%, only four masses (4%) were classified as benign and would have received surveillance, had the rule been applied. Thus, the clinical relevance of the rule was limited. Three of the four masses were indeed completely benign. Of the 101 masses, 77 (76%) considered as tumour actually contained tumour. Some 84 of the 241 masses, which were not resected (35%) had predicted probabilities of benign histology under 70% and would be considered as tumour.

## DISCUSSION

This study shows a reasonable statistical performance of the ReHiT prediction rule for residual mass histology of nonseminomatous testicular cancer in 105 recently treated patients with good prognosis disease. However, the clinical relevance of the rule was disappointing for these patients.

The prevalence of benign masses was low, that is, 26% in contrast to 45% in the development population. This may seem surprising, since we only considered patients with good prognosis disease. However, the studied patients were a selection of all good prognosis patients. Predominantly, patients with residual masses larger than 10 mm were candidates for resection and included in the validation population (92% *vs* 70% of the development population). It is well-known that small masses are more often benign. If more good prognosis patients with very small masses had undergone resection, the proportion of benign residual masses would have been higher.

In total, 30% of all patients (32 out of 105) had larger masses after chemotherapy than before, compared with 8% of the patients in the development population. Ignoring the 13 masses that were enlarged by less than 25% (which may simply reflect measurement error) reduces the proportion of enlarged masses to 18% (19 out of 105).

Low predicted probabilities showed disagreement with the observed frequencies, while higher predicted probabilities were well calibrated (Figure 2). Since a physician will choose surveillance over resection only if the predicted probability for benign histology is relatively high, the rule can still be valuable in that decision-making process. A larger sample size would, however, be required to provide solid evidence of adequate calibration.

Discriminative ability depends, apart from the studied model, also on the patients to whom the model is applied. If the predictor values of the patients show little variability (homogeneous population), it is difficult to distinguish between patients with different outcomes. Therefore, an ROC area of 0.76 is considered reasonable for our more homogeneous validation population containing only patients with good prognosis disease. A model with the same six predictor variables developed with the validation data resulted in a slightly larger ROC area (0.78). This confirms the finding that the original model was statistically valid for the good prognosis patients, even though the small sample size and the large confidence interval of the ROC area leave some room for doubt.

If a threshold value of 70% was used for the present patients, only four patients (4%) would be classified as benign. Thus, surveillance would be chosen over resection for 4% of the resected patients. Therefore, application of the model would have little clinical relevance for the present candidates of resection.

We also studied the clinical relevance of simpler models. If all patients with masses ⩽10 mm were to be offered surveillance, eight patients would have been denied resection of whom five had tumour. Considering mass size (⩽10 mm) together with the primary tumour histology (mature teratoma elements absent) would have resulted in only two patients being offered surveillance of whom one still had tumour. This suggests that simpler models are not to constitute good alternatives in good prognosis patients. Better discriminating selection models are required, to reduce the morbidity of treatment in these patients.

One-third of the patients who did not undergo resection because of small residual masses had predicted probabilities of benign histology under 70%, which indicates a substantial risk for residual tumour. A number of these patients should have been candidates for resection, particularly since the risks of short-term morbidities associated with resection are probably low given the size of the residual masses (Gels *et al*, 1997). The patients mainly had mature teratoma-positive primaries, elevated prechemotherapy levels of AFP or HCG or a low LDH level. Thus, the prediction rule could be particularly relevant in identifying small masses containing tumour. Future studies are required among patients currently offered surveillance to evaluate the role of the prediction rule.

To classify masses as benign or tumour using the prediction rule, we applied a threshold value of 70%. The assessment of a sensible threshold value is often difficult. We previously found that the policy to resect all masses larger than 10 mm had an implicit threshold value of 62% (Steyerberg *et al*, 1999b). A more stringent policy such as resection in all patients, except in those with masses smaller or equal to 20 mm, having a teratoma-negative primary tumour, and normal prechemotherapy levels of AFP and HCG (Fosså *et al*, 1992) implied a threshold value of 85%. A threshold value of 70 or 80% therefore seems reasonable.

Like any scientific hypothesis, the transportability of a prediction rule is established by being tested and being found valid across increasingly diverse settings (Justice *et al*, 1999). The more numerous and diverse the settings in which the rule is tested and found valid, the more likely it is that it will be transportable to an untested setting. Previously, we demonstrated the statistical performance of the prediction rule in a population of the late 1980s (Table 2

**Table 2:** Studies performed to validate the ReHiT prediction rule for nonseminomatous testicular cancer

	**Hospitals/groups**	**Years**	***n***	**Prognosis**	**Percentage benign masses**	**Calibration**	**Discrimination**	**Clinical relevance**
Development	Six study groups from	1979–92	544	Good/int/poor	45	OK	AUC=0.83	142/544 (26%) cons nec
	Europe and US							116/142 (82%) correct
Validation 1	Five study groups	1980–96	172	Good/int/poor	45	OK	AUC=0.82	52/172 (30%) cons nec
	from Europe							38/52 (73%) correct
Validation 2^a^	Indiana University	1985–99	276	Good/int/poor	28	Recalibration necessary	AUC=0.79	24/276 (9%) cons nec
								17/24 (71%) correct
Validation 3	EORTC/MRC trial	1995–98	105	Good	26	OK for predictions >5%	AUC=0.76	4/105 (4%) cons
(present study)								3/4 (75%) correct

Calibration=agreement between predicted probabilities and observed frequencies, Discrimination=ability to distinguish a benign mass from tumour, AUC=area under the curve, cons nec=masses considered as benign (predicted probability >70%), correct=masses correctly considered as benign (predicted probability >70% and histology benign).

a Modified prediction rule.

), which was rather similar to the development population (Steyerberg *et al*, 1998). The rule systematically predicted too high probabilities, for patients treated between 1985 and 1999 at Indiana University Medical Center (Vergouwe *et al*, 2001). For these patients, a simple adjustment of the prediction rule would result in better calibrated probabilities.

The rule was mainly clinically relevant for the patients from the development and first validation populations. Around 30% of the masses in these patients might have been considered benign and consequently would have been treated by surveillance. The clinical relevance was poor for the good prognosis patients from the present study (4% would have been treated by surveillance).

In conclusion, the prediction rule for residual mass histology is statistically valid in diverse settings. Given the small number of patients in the current study, the validity in good prognosis patients is still not fully certain. Although the clinical relevance was low for the resected patients, the rule may be valuable to identify candidates for resection among these with masses smaller than 10 mm containing tumour.
